# Syntheses and crystal structures of the one-dimensional coordination polymers formed by [Ni(cyclam)]^2+^ cations and 1,3-bis­(3-carb­oxy­prop­yl)tetra­methyl­disiloxane anions in different degrees of deprotonation

**DOI:** 10.1107/S2056989020002327

**Published:** 2020-02-25

**Authors:** Sergey P. Gavrish, Sergiu Shova, Maria Cazacu, Mihaela Dascalu, Yaroslaw D. Lampeka

**Affiliations:** a L.V. Pisarzhevskii Institute of Physical Chemistry of the National Academy of Sciences of Ukraine, Prospekt Nauki 31, Kyiv 03028, Ukraine; b "Petru Poni" Institute of Macromolecular Chemistry, Department of Inorganic Polymers, Aleea Grigore Ghica Voda 41A, RO-700487 Iasi, Romania

**Keywords:** crystal structure, macrocyclic ligand, cyclam, nickel, coordination polymers, hydrogen bonds

## Abstract

The asymmetric units of the title compounds, namely, *catena*-poly[[(1,4,8,11-tetra­aza­cyclo­tetra­decane-κ^4^ *N* ^1^,*N* ^4^,*N* ^8^,*N* ^11^)nickel(II)]-μ-1,3-bis­(3-carboxyl­ato­prop­yl)tetra­methyl­disiloxane-κ^2^ *O*:*O*′], [Ni(C_10_H_24_O_5_Si_2_)(C_12_H_24_N_4_)]_*n*_ (**I**), and *catena*-poly[[[(1,4,8,11-tetra­aza­cyclo­tetra­decane-κ^4^ *N* ^1^,*N* ^4^,*N* ^8^,*N* ^11^)nickel(II)]-μ-4-({[(3-carb­oxy­prop­yl)di­methyl­sil­yl]­oxy}di­methyl­sil­yl)butano­ato-κ^2^ *O*:*O*′] per­chlorate], {[Ni(C_10_H_25_O_5_Si_2_)(C_12_H_24_N_4_)]ClO_4_}_*n*_ (**II**), consist of one (in **I**) or two crystallographically non-equivalent (in **II**) centrosymmetric macrocyclic cations and one centrosymmetric dianion (in **I**) or two centrosymmetric monoanions (in **II**). In each compound, the metal ion is coordinated by the four secondary N atoms of the macrocyclic ligand, which adopts the most energetically stable *trans*-III conformation, and the mutually *trans* O atoms of the carboxyl­ate in a slightly tetra­gonally distorted *trans*-NiN_4_O_2_ octa­hedral coordination geometry. The crystals of both types of compounds are composed of parallel polymeric chains of the macrocyclic cations linked by the anions of the acid running along the [101] and [110] directions in **I** and **II**, respectively. In **I**, each polymeric chain is linked to four neighbouring ones by hydrogen bonding between the NH groups of the macrocycle and the carboxyl­ate O atoms, thus forming a three-dimensional supra­molecular network. In **II**, each polymeric chain contacts with only two neighbours, forming hydrogen bonds between the partially protonated carb­oxy­lic groups of the bridging ligand. As a result, a lamellar structure is formed with the layers oriented parallel to the (11) plane.

## Abstract 2

The title coordination polymers show different degrees of deprotonation of the disiloxane-di­carboxyl­ate bridging ligands: both contain tetra­gonally distorted *trans*-NiN_4_O_2_ octa­hedra.

## Chemical context   

Transition-metal complexes of polyaza­macrocyclic ligands, in particular of 1,4,8,11-tetra­aza­cyclo­tetra­decane (cyclam), are characterized by a number of unique properties, such as exceptionally high thermodynamic stability, kinetic inertness and unusual redox characteristics (Melson, 1979 ▸; Yatsimirskii & Lampeka, 1985 ▸), which have stimulated continuing inter­est in such systems for a number of decades. In conjunction with polycarboxyl­ate ligands as spacers, macrocyclic complexes have been employed successfully for the construction of metal–organic frameworks (MOFs) (Lampeka & Tsymbal, 2004 ▸; Suh & Moon, 2007 ▸; Suh *et al.*, 2012 ▸; Stackhouse & Ma, 2018 ▸), which are considered to be promising materials for applications in gas storage, separation, catalysis, *etc*. (Farrusseng, 2011 ▸; MacGillivray & Lukehart, 2014 ▸; Kaskel, 2016 ▸).

In contrast to the widespread rigid aromatic carboxyl­ates, flexible spacers incorporating polymethyl­ene chains have rarely been used for the design of MOFs, although this could potentially lead to frameworks possessing unusual properties, the most intriguing of which is a ‘breathing’ phenomenon (Elsaidi *et al.*, 2018 ▸; Lee *et al.*, 2019 ▸). A representative example of such a highly flexible ligand is 1,3-bis­(3-carb­oxy­prop­yl)tetra­methyl­disiloxane – a member of a rather restricted family of silicon-containing carb­oxy­lic acids. However, no attempt has been made so far to combine this ligand with macrocyclic complexes in MOF synthesis.

Here, we report the syntheses and crystal structures of the two coordination polymers built of the nickel(II) complex of the 14-membered macrocyclic ligand 1,4,8,11-tetra­aza­cyclo­tetra­decane (*L*) and the di- or monoanion of 1,3-bis(3-carb­oxy­prop­yl)tetra­methyl­disiloxane (H_2_Cx), namely, *catena*-poly[[(1,4,8,11-tetra­aza­cyclo­tetra­decane-κ^4^ *N* ^1^,*N* ^4^,*N* ^8^,*N* ^11^)nickel(II)]-μ-1,3-bis­(3-carboxyl­atoprop­yl)tetra­methyl­disiloxane-κ^2^ *O*:*O*′], [Ni(*L*)(Cx)]_*n*_, (**I**) and *catena*-poly[[[(1,4,8,11-tetra­aza­cyclo­tetra­decane-κ^4^ *N* ^1^,*N* ^4^,*N* ^8^,*N* ^11^)nickel(II)]-μ-4-({[(3-carb­oxy­prop­yl)di­methyl­sil­yl]­oxy}di­methyl­sil­yl)butano­ato-κ^2^ *O*:*O*′] perchlorate], {[Ni(*L*)(HCx)]ClO_4_}_*n*_ (**II**).
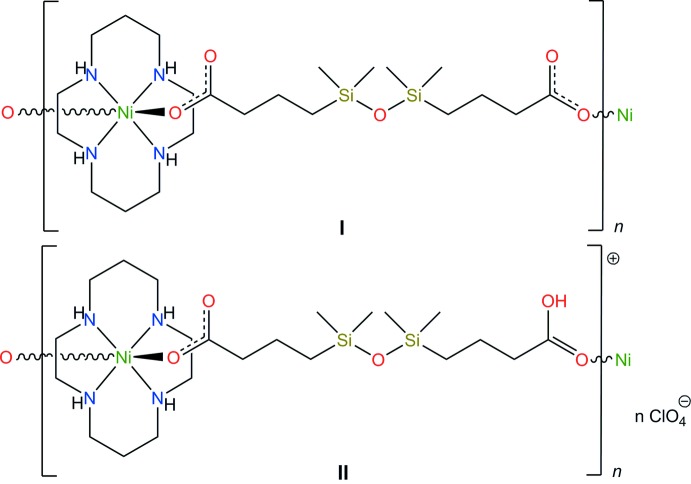


## Structural commentary   

**Figure 1:**
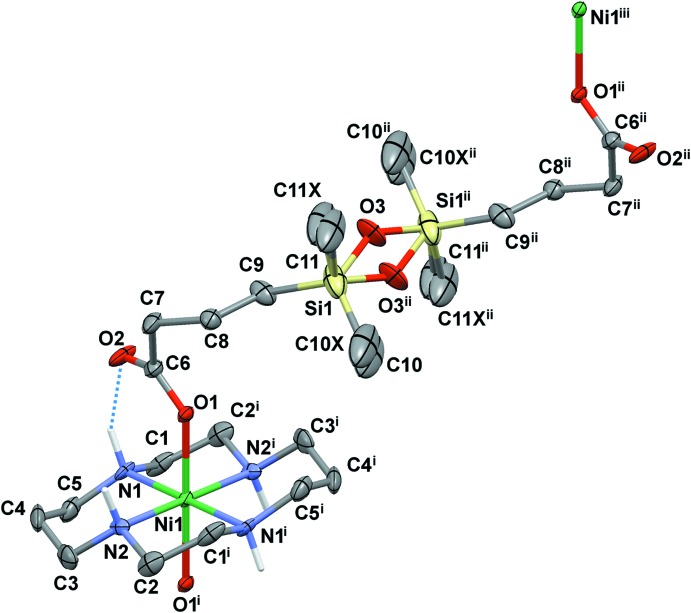
View of the mol­ecular structure of **I** showing atom-labelling scheme with displacement ellipsoids drawn at the 30% probability level. C-bound H atoms are omitted for clarity. Hydrogen-bonding inter­actions are shown as dotted lines. [Symmetry codes: (i) −*x* + 2, −*y* + 1, −*z* + 1; (ii) −*x* + 1, −*y* + 1, −*z*); (iii) *x* − 1, *y*, *z* − 1].

The mol­ecular structures of the title compounds are shown in Figs. 1 ▸ and 2 ▸. Both complexes are one-dimensional coordination polymers consisting of centrosymmetric macrocyclic [Ni(*L*)]^2+^ cations coordinated by the oxygen atoms of the carb­oxy­lic groups of the centrosymmetric acid, completely deprotonated (in **I**) and monoprotonated (in **II**), in the axial positions. In the latter case, there are two crystallographically independent cations and anions and the H2*C* and H5*C* acidic H atoms are distributed over two carb­oxy­lic groups with site occupancies of 50%.

The macrocyclic ligands in the complex cations adopt the most energetically favourable *trans*-III (*R,R,S,S*) conformation (Bosnich *et al.*, 1965 ▸) with five-membered chelate rings in *gauche* and six-membered chelate rings in *chair* conformations. As a result of the presence of the inversion centres, all Ni(N_4_) fragments are strictly planar. The equatorial Ni—N bond lengths and bite angles fall in a range typical of high-spin 3*d* ^8^ nickel(II) complexes with 14-membered tetra­amine ligands (Table 1 ▸). The axial Ni—O bond lengths are slightly longer than the Ni—N ones, and the geometry of the nickel(II) polyhedra can be described as tetra­gonally distorted *trans*-N_4_O_2_ octa­hedra.

In two cases (Ni1 in **I** and Ni2 in **II**), a monodentate coordination of the carboxyl­ate to the complex cation is complemented by strong hydrogen bonding between the non-coordinated O atom of the carb­oxy­lic group and the NH group of the macrocycle, which is often observed in complexes of cyclam-like ligands. For the [Ni1(*L*)]^2+^ cation in **II**, the non-coordinated O2 atom is almost equidistant from the N1 and N2 centres [3.225 (5) and 3.143 (4) Å, respectively], so that two weak hydrogen bonds are formed in this case (Figs. 1 ▸ and 2 ▸, Tables 2 ▸ and 3 ▸).

The C—O bond lengths in the carb­oxy­lic group of the bridging ligand Cx^2−^ in **I** are nearly identical [C6—O1 = 1.245 (7) and C6—O2 = 1.242 (7) Å], thus indicating essential electronic delocalization. At the same time, they differ significantly in **II** [C6—O1 = 1.232 (4) *versus* C6—O2 = 1.291 (5) Å; C17—O4 = 1.245 (4) *versus* C17—O5 = 1.280 (5) Å], so formally the Ni—O bonding in this compound can be treated as the inter­action of the metal ion with the carbonyl oxygen atom of the carb­oxy­lic group.

**Figure 3:**
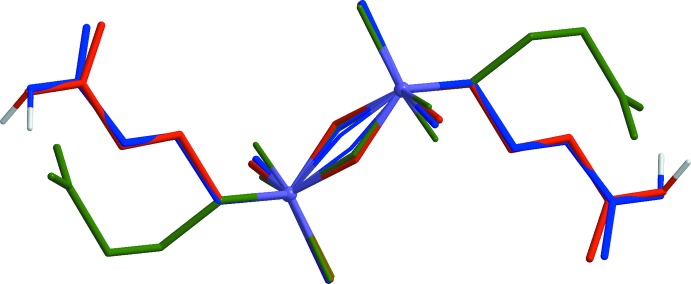
Comparison of the conformations of the dianion Cx^2−^ in **I** (green) and of the monoanions HCx^−^ in **II** (red and blue).

Because of the presence of flexible tri­methyl­ene fragments, the di­carboxyl­ate ligand can adopt various conformations, both symmetric and asymmetric. In the present cases the anions possess a transoid conformation of the siloxane linkages with the disordered O3 atoms [site occupancies 50%, Si1—O3—Si1 = 141.2 (7) and 137.4 (4)° in **I** and **II**, respectively], as well as with the 25% occupancy atoms O6 and O6*X* in **II** [the corresponding Si2—O6(6*X*)—Si2 angles are 153.1 (17) and 167 (3)°, respectively] (Figs. 1 ▸ and 2 ▸). The geometries of the two crystallographically independent anions in complex **II** are actually very similar, but differ from that observed in complex **I** (Fig. 3 ▸).

## Supra­molecular features   

**Figure 4:**
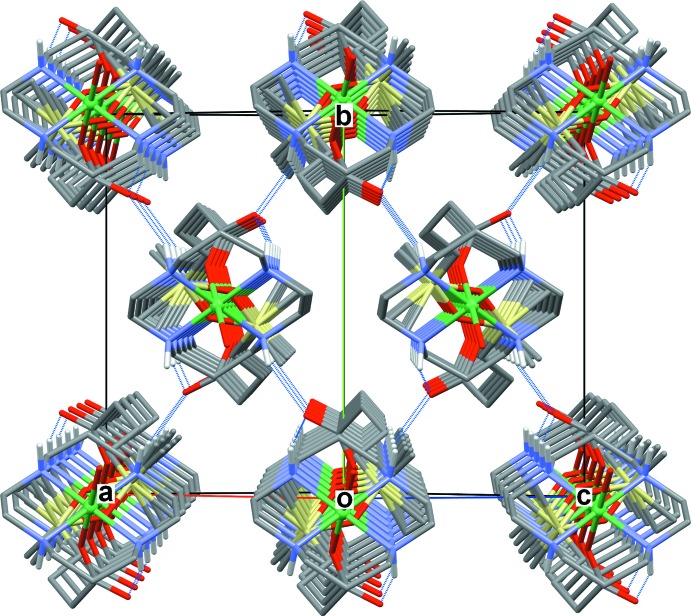
The packing in **I** viewed down the [101] direction with polymeric chains cross-linked by N—H⋯O hydrogen bonds (dotted lines) to form a three-dimensional supra­molecular network. C-bound H atoms are omitted for clarity.

**Figure 5:**
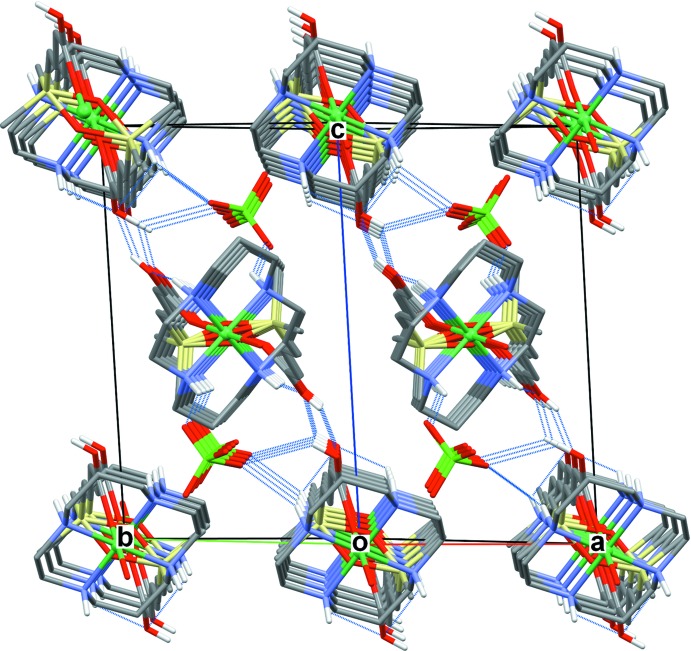
The packing in **II** viewed down the [110] direction with polymeric chains cross-linked by hydrogen bonds (dotted lines).

The crystals of both compounds are composed of parallel polymeric chains of [Ni(*L*)]^2+^ cations linked by carboxyl­ate bridging ligands. The identical chains in **I** with an intra-chain Ni⋯Ni separation of 14.325 Å propagate along the [101] direction (Fig. 4 ▸). In **II**, two crystallographically independent chains formed by the Ni1 and Ni2 macrocyclic cations propagate along the [110] direction (Fig. 5 ▸) and are characterized by a slightly larger (14.684 Å) intra-chain separation between the Ni^II^ ions.

**Figure 6:**
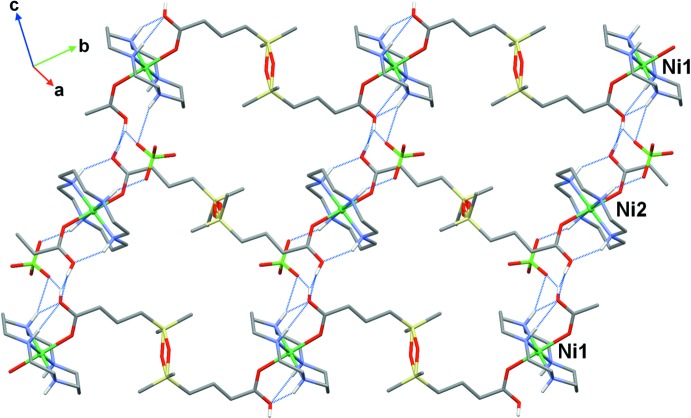
The hydrogen-bonded sheet in **II** parallel to the (1
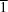
1) plane. C-bound H atoms are omitted for clarity.

In the crystals, the inter­actions between the polymeric chains in **I** and **II** are characterized by markedly different features. In the first case, each chain is linked to four neighbouring ones as a result of hydrogen bonding between the N2—H2 groups of the macrocycles and carboxyl­ate O2 atoms (Table 2 ▸), resulting in a three-dimensional supra­molecular network. On the other hand, in **II** each polymeric chain contacts with only two neighbours *via* paired O2—H2*C*⋯O5/O2⋯H5*C*—O5 hydrogen bonds. The bonding is reinforced by the perchlorate anions bridging macrocyclic units: N1—H1⋯O8—Cl1—O7⋯H4—N4 (plus an additional very weak O2—H2*C*⋯O8 contact) (Table 3 ▸). As a result, a lamellar structure is formed with the layers lying parallel to the (1
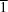
1) plane (Fig. 6 ▸).

## Database survey   

A search of the Cambridge Structural Database (CSD, version 5.40, last update February 2019; Groom *et al.*, 2016 ▸) indicated that seven compounds formed by 1,3-bis­(3-carb­oxy­prop­yl)tetra­methyl­disiloxane itself or its anions have been characterized structurally. Two of them are co-crystals of the acid with organic bases derived from pyridine [refcodes NERTOV (Vlad *et al.*, 2013*a* ▸) and VIPZUR (Racles *et al.*, 2013 ▸)]. Other complexes represent one- or two-dimensional coordination polymers formed by Cu^II^ (YIGXOD; Vlad *et al.*, 2013*b* ▸), Co^II^ (NERTIP; Vlad *et al.*, 2013*a* ▸), Zn^II^ [NERTUB (Vlad *et al.*, 2013*a* ▸), GIWSAI (Vlad *et al.*, 2014 ▸) and GAPKOA (Zaltariov *et al.*, 2016 ▸)]. Except for the last complex, in which the secondary building unit is a hexa­metal oxocluster bridged by salicylaldoxime ligands, all of the other compounds contain additional heterocyclic co-ligands. No attempt was made to combine this carb­oxy­lic acid with macrocyclic cations in MOF synthesis, and thus the title compounds **I** and **II** are the first examples of such compounds described so far.

## Synthesis and crystallization   

All chemicals and solvents used in this work were purchased from Sigma–Aldrich and were used without further purification. The macrocyclic nickel(II) complex Ni(*L*)(ClO_4_)_2_ (Barefield *et al.*, 1976 ▸) and 1,3-bis­(3-carb­oxy­prop­yl)tetra­methyl­disiloxane (H_2_Cx) (Mulvaney & Marvel, 1961 ▸) were prepared by the reported methods.

**{Ni(** ***L*** **)(Cx)}** ***_n_***, (**I**). To a solution of 48 mg (0.24 mmol) of the ligand *L* in 4 ml of water, 30 mg of nickel(II) hydroxide (0.32 mmol) were added and the suspension stirred for 4 d at room temperature to give a yellow-coloured solution. The excess of Ni(OH)_2_ was filtered off and the filtrate was treated with the solution of 75 mg (0.24 mmol) of H_2_Cx in 2 ml of MeOH. This solution was rotary evaporated to give an oily material. The residue was dissolved in 2 ml of MeOH, and the product precipitated with aceto­nitrile. It was recrystallized in a similar fashion from a MeOH/MeCN (1:15 *v*/*v*) solvent mixture. Yield 54 mg (40%). Analysis calculated for C_22_H_48_N_4_NiO_5_Si_2_: C, 46.89; H, 8.59; N, 9.94%. Found: C, 46.76; H, 8.64; N, 9.85%.

Single crystals of **I** suitable for X-ray diffraction analysis were obtained analogously, except that precipitation was carried out using a diffusion regime (a methano­lic solution of complex was layered with MeCN).

**{[Ni(** ***L*** **)(HCx)]ClO_4_}** ***_n_*** **(II)**. A solution of 100 mg (0.26 mmol) of K_2_Cx in 1 ml of water was added to a solution of 130 mg (0.28 mmol) of [Ni(*L*)](ClO_4_)_2_ in 3 ml of water and the mixture was left at room temperature. Potassium perchlorate crystals, which formed after *ca* two weeks, were removed by filtration and the filtrate was allowed to evaporate slowly at room temperature. The crystals of the product formed after about one month. Yield 59 mg (34%). Analysis calculated for C_22_H_49_N_4_ClNiO_9_Si_2_: C, 39.80; H, 7.44; N, 8.44%. Found: C, 39.67; H, 7.51; N, 8.36%.

Single crystals of **II** suitable for X-ray diffraction analysis were selected from the sample resulting from the synthesis.

**Safety note**: Perchlorate salts of metal complexes are potentially explosive and should be handled with care.

## Refinement   

Crystal data, data collection and structure refinement details are summarized in Table 4 ▸. All H atoms in **I** and **II** were placed in geometrically idealized positions and constrained to ride on their parent atoms, with C—H = 0.97 Å, N—H = 0.98 Å and carboxyl­ate O—H = 0.82 Å, with *U*_iso_(H) values of 1.2 or 1.5*U*_eq_ of the parent atoms.

## Supplementary Material

Crystal structure: contains datablock(s) I, II. DOI: 10.1107/S2056989020002327/hb7892sup1.cif

Structure factors: contains datablock(s) I. DOI: 10.1107/S2056989020002327/hb7892Isup2.hkl

Structure factors: contains datablock(s) II. DOI: 10.1107/S2056989020002327/hb7892IIsup3.hkl

CCDC references: 1985068, 1985067

Additional supporting information:  crystallographic information; 3D view; checkCIF report

## Figures and Tables

**Figure 2:**
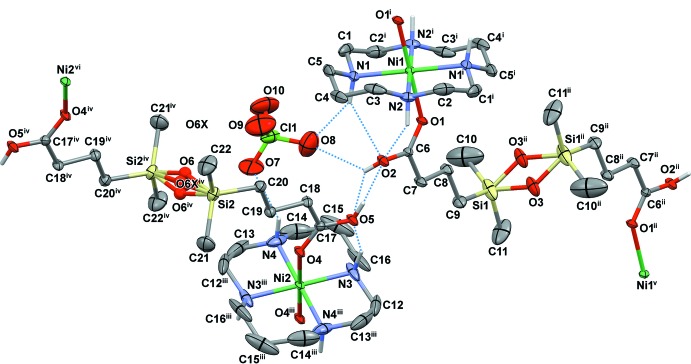
View of the mol­ecular structure of **II** showing atom-labelling scheme with displacement ellipsoids drawn at the 30% probability level. C-bound H atoms are omitted for clarity. Hydrogen-bonding inter­actions are shown as dotted lines. [Symmetry codes: (i) −*x*, −*y*, −*z*; (ii) −*x* − 1, −*y* − 1, −*z*; (iii) −*x*, −*y* − 1, −*z* − 1; (iv) −*x* + 1, −*y*, −*z* − 1; (v) *x* − 1, *y* − 1, *z*; (vi) *x* + 1, *y* + 1, *z*.]

**Table 1:** Selected geometrical parameters of the complex cations (Å, °)

**I**		**II**			
Ni1—N1	2.071 (4)	Ni1—N1	2.058 (3)	Ni2—N3	2.043 (4)
Ni1—N2	2.060 (4)	Ni1—N2	2.060 (4)	Ni2—N4	2.054 (4)
Ni1—O1	2.113 (4)	Ni1—O1	2.125 (2)	Ni2—O4	2.131 (2)
					
N1—Ni1—N2^i^	85.21 (19)	N1—Ni1—N2^ii^	85.82 (17)	N3—Ni2—N4^iii^	85.7 (2)
N1—Ni1—N2	94.79 (19)	N1—Ni1—N2	94.18 (17)	N3—Ni2—N4	94.3 (2)

Symmetry codes: (i) −*x* + 2, −*y* + 1, −*z* + 1; (ii) −*x*, −*y*, −*z*; (iii) −*x*, −*y* − 1, −*z* − 1.

**Table 2:** Hydrogen-bond geometry (Å, °) for **I** [Chem scheme1]

*D*—H⋯*A*	*D*—H	H⋯*A*	*D*⋯*A*	*D*—H⋯*A*
N1—H1⋯O2	0.98	1.96	2.845 (6)	150
N2—H2⋯O2^i^	0.98	2.07	2.883 (6)	139

Symmetry code: (i) 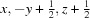.

**Table 3:** Hydrogen-bond geometry (Å, °) for **II** [Chem scheme1]

*D*—H⋯*A*	*D*—H	H⋯*A*	*D*⋯*A*	*D*—H⋯*A*
N1—H1⋯O2	0.98	2.51	3.225 (5)	130
N1—H1⋯O8	0.98	2.45	3.315 (6)	147
N2—H2⋯O2	0.98	2.38	3.143 (4)	134
N3—H3⋯O5	0.98	2.01	2.901 (5)	150
N4—H4⋯O7	0.98	2.18	3.012 (6)	142
O2—H2*C*⋯O5	0.82	1.84	2.456 (4)	131
O2—H2*C*⋯O8	0.82	2.65	3.260 (5)	133
O5—H5*C*⋯O2	0.82	1.70	2.456 (4)	151

**Table 4:** Experimental details

	**I**	**II**
Crystal data		
Chemical formula	[Ni(C_10_H_24_O_5_Si_2_)(C_12_H_24_N_4_)]	[Ni(C_10_H_25_O_5_Si_2_)(C_12_H_24_N_4_)]ClO_4_
*M*_r_	563.53	663.99
Crystal system, space group	Monoclinic, *P*2_1_/*c*	Triclinic, *P* 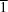
Temperature (K)	173	200
*a*, *b*, *c* (Å)	13.033 (5), 12.877 (10), 9.028 (3)	9.3815 (7), 12.9009 (8), 14.7604 (10)
α, β, γ (°)	90, 101.31 (3), 90	99.309 (5), 100.343 (6), 99.232 (6)
*V* (Å^3^)	1485.7 (13)	1700.9 (2)
*Z*	2	2
Radiation type	Mo *K*α	Mo *K*α
μ (mm^−1^)	0.77	0.77
Crystal size (mm)	0.25 × 0.25 × 0.05	0.45 × 0.35 × 0.30

Data collection		
Diffractometer	Agilent Xcalibur, Eos	Agilent Xcalibur, Eos
Absorption correction	Multi-scan (*CrysAlis PRO*; Agilent, 2014 ▸)	Multi-scan (*CrysAlis PRO*; Agilent, 2014 ▸)
*T*_min_, *T*_max_	0.694, 1.000	0.889, 1.000
No. of measured, independent and observed [*I* > 2σ(*I*)] reflections	3957, 3957, 2499	9606, 9606, 5769
*R*_int_	0.040	0.063
(sin θ/λ)_max_ (Å^−1^)	0.595	0.595

Refinement		
*R*[*F* ^2^ > 2σ(*F* ^2^)], *wR*(*F* ^2^), *S*	0.065, 0.143, 1.01	0.050, 0.115, 1.01
No. of reflections	3957	9606
No. of parameters	165	367
No. of restraints	6	7
H-atom treatment	H-atom parameters constrained	H-atom parameters constrained
Δρ_max_, Δρ_min_ (e Å^−3^)	0.56, −0.61	0.51, −0.44

Computer programs: *CrysAlis PRO* (Agilent, 2014 ▸), *SIR2008* (Burla *et al.*, 2007 ▸), *SHELXT* (Sheldrick, 2015*a* ▸), *SHELXL2018/3* (Sheldrick, 2015*b* ▸), *Mercury* (Macrae *et al.*, 2020 ▸) and *publCIF* (Westrip, 2010 ▸).

## supplementary crystallographic information

### catena-Poly[[(1,4,8,11-tetraazacyclotetradecane-κ4N1,N4,N8,N11)nickel(II)]-µ-1,3-bis(3-carboxylatopropyl)tetramethyldisiloxane-κ2O:O'] (I) Crystal data

**Table:**

[Ni(C_10_H_24_O_5_Si_2_)(C_12_H_24_N_4_)]	*F*(000) = 608
*M**_r_* = 563.53	*D*_x_ = 1.260 Mg m^−^^3^
Monoclinic, *P*2_1_/*c*	Mo *K*α radiation, λ = 0.71073 Å
*a* = 13.033 (5) Å	Cell parameters from 468 reflections
*b* = 12.877 (10) Å	θ = 2.2–23.0°
*c* = 9.028 (3) Å	µ = 0.77 mm^−^^1^
β = 101.31 (3)°	*T* = 173 K
*V* = 1485.7 (13) Å^3^	Plate, clear light colourless
*Z* = 2	0.25 × 0.25 × 0.05 mm

### catena-Poly[[(1,4,8,11-tetraazacyclotetradecane-κ4N1,N4,N8,N11)nickel(II)]-µ-1,3-bis(3-carboxylatopropyl)tetramethyldisiloxane-κ2O:O'] (I) Data collection

**Table:**

Agilent Xcalibur, Eos diffractometer	3957 independent reflections
Radiation source: Enhance (Mo) X-ray Source	2499 reflections with *I* > 2σ(*I*)
Graphite monochromator	*R*_int_ = 0.040
Detector resolution: 16.1593 pixels mm^-1^	θ_max_ = 25.0°, θ_min_ = 2.3°
ω scans	*h* = −15→15
Absorption correction: multi-scan (CrysAlis PRO; Agilent, 2014)	*k* = −15→15
*T*_min_ = 0.694, *T*_max_ = 1.000	*l* = −10→9
3957 measured reflections	

### catena-Poly[[(1,4,8,11-tetraazacyclotetradecane-κ4N1,N4,N8,N11)nickel(II)]-µ-1,3-bis(3-carboxylatopropyl)tetramethyldisiloxane-κ2O:O'] (I) Refinement

**Table:**

Refinement on *F*^2^	Primary atom site location: structure-invariant direct methods
Least-squares matrix: full	Hydrogen site location: inferred from neighbouring sites
*R*[*F*^2^ > 2σ(*F*^2^)] = 0.065	H-atom parameters constrained
*wR*(*F*^2^) = 0.143	*w* = 1/[σ^2^(*F*_o_^2^) + (0.0618*P*)^2^] where *P* = (*F*_o_^2^ + 2*F*_c_^2^)/3
*S* = 1.00	(Δ/σ)_max_ < 0.001
3957 reflections	Δρ_max_ = 0.56 e Å^−^^3^
165 parameters	Δρ_min_ = −0.61 e Å^−^^3^
6 restraints	

### catena-Poly[[(1,4,8,11-tetraazacyclotetradecane-κ4N1,N4,N8,N11)nickel(II)]-µ-1,3-bis(3-carboxylatopropyl)tetramethyldisiloxane-κ2O:O'] (I) Special details

**Table:**

Geometry. All esds (except the esd in the dihedral angle between two l.s. planes) are estimated using the full covariance matrix. The cell esds are taken into account individually in the estimation of esds in distances, angles and torsion angles; correlations between esds in cell parameters are only used when they are defined by crystal symmetry. An approximate (isotropic) treatment of cell esds is used for estimating esds involving l.s. planes.
Refinement. Refined as a 2-component twin.

### catena-Poly[[(1,4,8,11-tetraazacyclotetradecane-κ4N1,N4,N8,N11)nickel(II)]-µ-1,3-bis(3-carboxylatopropyl)tetramethyldisiloxane-κ2O:O'] (I) Fractional atomic coordinates and isotropic or equivalent isotropic displacement parameters (Å^2^)

**Table:**

	*x*	*y*	*z*	*U*_iso_*/*U*_eq_	Occ. (<1)
Ni1	1.000000	0.500000	0.500000	0.0224 (3)	
Si1	0.51530 (16)	0.4465 (2)	0.1590 (4)	0.0810 (8)	
O1	0.8723 (3)	0.4020 (3)	0.4131 (4)	0.0278 (10)	
O2	0.9375 (4)	0.2757 (3)	0.2920 (5)	0.0546 (13)	
O3	0.4991 (12)	0.4662 (10)	−0.0400 (11)	0.090 (4)	0.5
N1	1.1059 (4)	0.4111 (4)	0.4113 (5)	0.0313 (13)	
H1	1.068906	0.348403	0.368383	0.038*	
N2	1.0173 (4)	0.4173 (4)	0.6987 (5)	0.0302 (12)	
H2	0.974805	0.354292	0.677540	0.036*	
C1	1.1301 (5)	0.4718 (5)	0.2850 (7)	0.047 (2)	
H1A	1.161336	0.427243	0.219319	0.057*	
H1B	1.179620	0.526309	0.323016	0.057*	
C2	0.9705 (5)	0.4810 (5)	0.8028 (7)	0.050 (2)	
H2A	1.018601	0.535473	0.845711	0.060*	
H2B	0.955552	0.438364	0.884604	0.060*	
C3	1.1239 (5)	0.3849 (5)	0.7630 (7)	0.051 (2)	
H3A	1.123929	0.344748	0.853931	0.061*	
H3B	1.167199	0.445811	0.790758	0.061*	
C4	1.1691 (6)	0.3211 (6)	0.6537 (9)	0.056 (2)	
H4A	1.118692	0.267578	0.614158	0.067*	
H4B	1.230881	0.286356	0.709093	0.067*	
C5	1.1991 (5)	0.3767 (5)	0.5216 (8)	0.050 (2)	
H5A	1.241788	0.436626	0.557967	0.061*	
H5B	1.240623	0.330662	0.471842	0.061*	
C6	0.8685 (5)	0.3154 (5)	0.3510 (7)	0.0354 (16)	
C7	0.7696 (5)	0.2514 (5)	0.3470 (8)	0.0449 (18)	
H7A	0.778986	0.208718	0.437187	0.054*	
H7B	0.760544	0.205156	0.260604	0.054*	
C8	0.6702 (5)	0.3156 (5)	0.3376 (7)	0.0403 (18)	
H8A	0.613522	0.269978	0.350886	0.048*	
H8B	0.680861	0.365496	0.419680	0.048*	
C9	0.6386 (4)	0.3731 (5)	0.1896 (8)	0.0517 (19)	
H9A	0.634154	0.323019	0.108349	0.062*	
H9B	0.694402	0.421166	0.180578	0.062*	
C10X	0.4972 (14)	0.5342 (14)	0.313 (2)	0.139 (5)	0.5
H10A	0.504595	0.495602	0.405513	0.209*	0.5
H10B	0.428590	0.564443	0.289455	0.209*	0.5
H10C	0.548903	0.588187	0.324362	0.209*	0.5
C11	0.3986 (16)	0.369 (2)	0.171 (7)	0.139 (5)	0.5
H11A	0.340588	0.414111	0.173486	0.209*	0.5
H11B	0.412287	0.327371	0.261206	0.209*	0.5
H11C	0.382182	0.324019	0.084404	0.209*	0.5
C10	0.5355 (15)	0.5612 (13)	0.283 (2)	0.139 (5)	0.5
H10D	0.581626	0.609001	0.247371	0.209*	0.5
H10E	0.565898	0.539997	0.384236	0.209*	0.5
H10F	0.469460	0.594367	0.282739	0.209*	0.5
C11X	0.4087 (17)	0.3521 (18)	0.156 (7)	0.139 (5)	0.5
H11D	0.342613	0.385730	0.121176	0.209*	0.5
H11E	0.411408	0.325433	0.255871	0.209*	0.5
H11F	0.416537	0.296006	0.088976	0.209*	0.5

### catena-Poly[[(1,4,8,11-tetraazacyclotetradecane-κ4N1,N4,N8,N11)nickel(II)]-µ-1,3-bis(3-carboxylatopropyl)tetramethyldisiloxane-κ2O:O'] (I) Atomic displacement parameters (Å^2^)

**Table:**

	*U*^11^	*U*^22^	*U*^33^	*U*^12^	*U*^13^	*U*^23^
Ni1	0.0285 (5)	0.0157 (5)	0.0236 (5)	0.0026 (6)	0.0068 (5)	0.0002 (6)
Si1	0.0406 (13)	0.0755 (19)	0.119 (2)	−0.0021 (12)	−0.0034 (15)	0.0400 (17)
O1	0.029 (2)	0.019 (3)	0.035 (3)	0.003 (2)	0.0052 (18)	−0.0074 (19)
O2	0.046 (3)	0.031 (3)	0.091 (4)	−0.006 (2)	0.023 (3)	−0.033 (3)
O3	0.103 (8)	0.105 (14)	0.043 (9)	0.010 (10)	−0.031 (9)	0.010 (6)
N1	0.037 (3)	0.014 (3)	0.046 (3)	−0.002 (3)	0.017 (3)	−0.012 (2)
N2	0.045 (3)	0.025 (3)	0.019 (3)	−0.013 (3)	0.001 (2)	0.005 (2)
C1	0.070 (5)	0.028 (5)	0.056 (5)	−0.014 (4)	0.042 (4)	−0.013 (3)
C2	0.077 (6)	0.039 (6)	0.040 (4)	−0.013 (4)	0.026 (4)	0.003 (4)
C3	0.060 (5)	0.046 (5)	0.040 (4)	−0.002 (4)	−0.007 (4)	0.023 (4)
C4	0.041 (4)	0.029 (5)	0.089 (6)	0.009 (4)	−0.009 (4)	0.018 (4)
C5	0.038 (4)	0.029 (5)	0.085 (6)	0.006 (4)	0.016 (4)	−0.008 (4)
C6	0.034 (4)	0.025 (5)	0.046 (4)	−0.002 (3)	0.003 (3)	−0.003 (3)
C7	0.038 (4)	0.022 (5)	0.074 (5)	−0.001 (3)	0.009 (3)	−0.008 (4)
C8	0.032 (4)	0.039 (5)	0.050 (4)	−0.002 (3)	0.007 (3)	−0.001 (3)
C9	0.034 (4)	0.067 (5)	0.053 (4)	−0.009 (4)	0.006 (4)	0.011 (4)
C10X	0.039 (6)	0.077 (7)	0.295 (14)	0.005 (5)	0.016 (7)	0.004 (9)
C11	0.039 (6)	0.077 (7)	0.295 (14)	0.005 (5)	0.016 (7)	0.004 (9)
C10	0.039 (6)	0.077 (7)	0.295 (14)	0.005 (5)	0.016 (7)	0.004 (9)
C11X	0.039 (6)	0.077 (7)	0.295 (14)	0.005 (5)	0.016 (7)	0.004 (9)

### catena-Poly[[(1,4,8,11-tetraazacyclotetradecane-κ4N1,N4,N8,N11)nickel(II)]-µ-1,3-bis(3-carboxylatopropyl)tetramethyldisiloxane-κ2O:O'] (I) Geometric parameters (Å, º)

**Table:**

Ni1—O1	2.113 (4)	C3—H3B	0.9700
Ni1—O1^i^	2.113 (4)	C3—C4	1.491 (9)
Ni1—N1	2.071 (4)	C4—H4A	0.9700
Ni1—N1^i^	2.071 (4)	C4—H4B	0.9700
Ni1—N2	2.060 (4)	C4—C5	1.508 (9)
Ni1—N2^i^	2.060 (4)	C5—H5A	0.9700
Si1—O3	1.785 (11)	C5—H5B	0.9700
Si1—O3^ii^	1.541 (13)	C6—C7	1.524 (9)
Si1—C9	1.838 (6)	C7—H7A	0.9700
Si1—C10X	1.842 (7)	C7—H7B	0.9700
Si1—C11	1.842 (7)	C7—C8	1.525 (8)
Si1—C10	1.842 (7)	C8—H8A	0.9700
Si1—C11X	1.842 (7)	C8—H8B	0.9700
O1—C6	1.245 (7)	C8—C9	1.512 (8)
O2—C6	1.242 (7)	C9—H9A	0.9700
O3—O3^ii^	1.13 (2)	C9—H9B	0.9700
N1—H1	0.9800	C10X—H10A	0.9600
N1—C1	1.467 (7)	C10X—H10B	0.9600
N1—C5	1.479 (7)	C10X—H10C	0.9600
N2—H2	0.9800	C11—H11A	0.9600
N2—C2	1.467 (7)	C11—H11B	0.9600
N2—C3	1.459 (7)	C11—H11C	0.9600
C1—H1A	0.9700	C10—H10D	0.9600
C1—H1B	0.9700	C10—H10E	0.9600
C1—C2^i^	1.519 (8)	C10—H10F	0.9600
C2—H2A	0.9700	C11X—H11D	0.9600
C2—H2B	0.9700	C11X—H11E	0.9600
C3—H3A	0.9700	C11X—H11F	0.9600
			
O1^i^—Ni1—O1	180.0	N2—C3—C4	111.3 (5)
N1—Ni1—O1	93.58 (17)	H3A—C3—H3B	108.0
N1^i^—Ni1—O1	86.42 (17)	C4—C3—H3A	109.4
N1—Ni1—O1^i^	86.42 (17)	C4—C3—H3B	109.4
N1^i^—Ni1—O1^i^	93.58 (17)	C3—C4—H4A	108.0
N1—Ni1—N1^i^	180.0	C3—C4—H4B	108.0
N2^i^—Ni1—O1	92.40 (16)	C3—C4—C5	117.3 (6)
N2—Ni1—O1^i^	92.40 (16)	H4A—C4—H4B	107.2
N2—Ni1—O1	87.60 (16)	C5—C4—H4A	108.0
N2^i^—Ni1—O1^i^	87.60 (16)	C5—C4—H4B	108.0
N2—Ni1—N1^i^	85.21 (19)	N1—C5—C4	111.6 (5)
N2^i^—Ni1—N1^i^	94.79 (19)	N1—C5—H5A	109.3
N2—Ni1—N1	94.79 (19)	N1—C5—H5B	109.3
N2^i^—Ni1—N1	85.21 (19)	C4—C5—H5A	109.3
N2^i^—Ni1—N2	180.0 (2)	C4—C5—H5B	109.3
O3^ii^—Si1—O3	38.8 (7)	H5A—C5—H5B	108.0
O3—Si1—C9	98.7 (5)	O1—C6—C7	117.0 (6)
O3^ii^—Si1—C9	117.6 (6)	O2—C6—O1	126.5 (6)
O3^ii^—Si1—C10X	93.6 (8)	O2—C6—C7	116.5 (6)
O3—Si1—C10X	131.7 (9)	C6—C7—H7A	108.7
O3—Si1—C11	102 (2)	C6—C7—H7B	108.7
O3^ii^—Si1—C11	116.7 (16)	C6—C7—C8	114.4 (5)
O3—Si1—C10	118.2 (9)	H7A—C7—H7B	107.6
O3^ii^—Si1—C10	79.8 (9)	C8—C7—H7A	108.7
O3—Si1—C11X	98 (2)	C8—C7—H7B	108.7
O3^ii^—Si1—C11X	118.8 (18)	C7—C8—H8A	108.9
C9—Si1—C10X	116.1 (7)	C7—C8—H8B	108.9
C9—Si1—C11	114.8 (10)	H8A—C8—H8B	107.7
C9—Si1—C10	107.7 (7)	C9—C8—C7	113.3 (5)
C9—Si1—C11X	107.3 (9)	C9—C8—H8A	108.9
C11—Si1—C10X	93.4 (18)	C9—C8—H8B	108.9
C11X—Si1—C10	123.8 (18)	Si1—C9—H9A	107.9
C6—O1—Ni1	131.4 (4)	Si1—C9—H9B	107.9
Si1^ii^—O3—Si1	141.2 (7)	C8—C9—Si1	117.6 (4)
O3^ii^—O3—Si1	58.8 (11)	C8—C9—H9A	107.9
O3^ii^—O3—Si1^ii^	82.4 (14)	C8—C9—H9B	107.9
Ni1—N1—H1	107.2	H9A—C9—H9B	107.2
C1—N1—Ni1	105.5 (4)	Si1—C10X—H10A	109.5
C1—N1—H1	107.2	Si1—C10X—H10B	109.5
C1—N1—C5	114.1 (5)	Si1—C10X—H10C	109.5
C5—N1—Ni1	115.3 (4)	H10A—C10X—H10B	109.5
C5—N1—H1	107.2	H10A—C10X—H10C	109.5
Ni1—N2—H2	107.3	H10B—C10X—H10C	109.5
C2—N2—Ni1	106.3 (4)	Si1—C11—H11A	109.5
C2—N2—H2	107.3	Si1—C11—H11B	109.5
C3—N2—Ni1	115.3 (4)	Si1—C11—H11C	109.5
C3—N2—H2	107.3	H11A—C11—H11B	109.5
C3—N2—C2	112.9 (5)	H11A—C11—H11C	109.5
N1—C1—H1A	109.9	H11B—C11—H11C	109.5
N1—C1—H1B	109.9	Si1—C10—H10D	109.5
N1—C1—C2^i^	108.9 (5)	Si1—C10—H10E	109.5
H1A—C1—H1B	108.3	Si1—C10—H10F	109.5
C2^i^—C1—H1A	109.9	H10D—C10—H10E	109.5
C2^i^—C1—H1B	109.9	H10D—C10—H10F	109.5
N2—C2—C1^i^	108.3 (5)	H10E—C10—H10F	109.5
N2—C2—H2A	110.0	Si1—C11X—H11D	109.5
N2—C2—H2B	110.0	Si1—C11X—H11E	109.5
C1^i^—C2—H2A	110.0	Si1—C11X—H11F	109.5
C1^i^—C2—H2B	110.0	H11D—C11X—H11E	109.5
H2A—C2—H2B	108.4	H11D—C11X—H11F	109.5
N2—C3—H3A	109.4	H11E—C11X—H11F	109.5
N2—C3—H3B	109.4		
			
Ni1—O1—C6—O2	−18.8 (10)	C6—C7—C8—C9	67.1 (7)
Ni1—O1—C6—C7	161.1 (4)	C7—C8—C9—Si1	176.0 (4)
Ni1—N1—C1—C2^i^	−41.9 (5)	C9—Si1—O3—Si1^ii^	−123.8 (15)
Ni1—N1—C5—C4	53.8 (7)	C9—Si1—O3—O3^ii^	−123.8 (15)
Ni1—N2—C2—C1^i^	41.2 (5)	C10X—Si1—O3—Si1^ii^	13 (2)
Ni1—N2—C3—C4	−57.1 (7)	C10X—Si1—O3—O3^ii^	13 (2)
O1—C6—C7—C8	31.8 (8)	C10X—Si1—C9—C8	49.5 (9)
O2—C6—C7—C8	−148.4 (6)	C11—Si1—O3—Si1^ii^	118.5 (18)
O3^ii^—Si1—O3—Si1^ii^	0.003 (1)	C11—Si1—O3—O3^ii^	118.5 (18)
O3—Si1—C9—C8	−165.0 (7)	C11—Si1—C9—C8	−58 (2)
O3^ii^—Si1—C9—C8	159.0 (6)	C10—Si1—O3—Si1^ii^	−8.3 (19)
N2—C3—C4—C5	72.9 (8)	C10—Si1—O3—O3^ii^	−8.3 (19)
C1—N1—C5—C4	176.2 (5)	C10—Si1—C9—C8	71.5 (10)
C2—N2—C3—C4	−179.6 (5)	C11X—Si1—O3—Si1^ii^	127.1 (18)
C3—N2—C2—C1^i^	168.6 (5)	C11X—Si1—O3—O3^ii^	127.1 (18)
C3—C4—C5—N1	−71.1 (8)	C11X—Si1—C9—C8	−64 (2)
C5—N1—C1—C2^i^	−169.5 (5)		

Symmetry codes: (i) −*x*+2, −*y*+1, −*z*+1; (ii) −*x*+1, −*y*+1, −*z*.

### catena-Poly[[(1,4,8,11-tetraazacyclotetradecane-κ4N1,N4,N8,N11)nickel(II)]-µ-1,3-bis(3-carboxylatopropyl)tetramethyldisiloxane-κ2O:O'] (I) Hydrogen-bond geometry (Å, º)

**Table:**

*D*—H···*A*	*D*—H	H···*A*	*D*···*A*	*D*—H···*A*
N1—H1···O2	0.98	1.96	2.845 (6)	150
N2—H2···O2^iii^	0.98	2.07	2.883 (6)	139

Symmetry code: (iii) *x*, −*y*+1/2, *z*+1/2.

### catena-Poly[[[(1,4,8,11-tetraazacyclotetradecane-κ4N1,N4,N8,N11)nickel(II)]-µ-4-({[(3-carboxypropyl)dimethylsilyl]oxy}dimethylsilyl)butanoato-κ2O:O'] perchlorate] (II) Crystal data

**Table:**

[Ni(C_10_H_25_O_5_Si_2_)(C_12_H_24_N_4_)]ClO_4_	*Z* = 2
*M**_r_* = 663.99	*F*(000) = 708
Triclinic, *P*1	*D*_x_ = 1.296 Mg m^−^^3^
*a* = 9.3815 (7) Å	Mo *K*α radiation, λ = 0.71073 Å
*b* = 12.9009 (8) Å	Cell parameters from 2033 reflections
*c* = 14.7604 (10) Å	θ = 1.7–24.6°
α = 99.309 (5)°	µ = 0.77 mm^−^^1^
β = 100.343 (6)°	*T* = 200 K
γ = 99.232 (6)°	Block, clear light colourless
*V* = 1700.9 (2) Å^3^	0.45 × 0.35 × 0.30 mm

### catena-Poly[[[(1,4,8,11-tetraazacyclotetradecane-κ4N1,N4,N8,N11)nickel(II)]-µ-4-({[(3-carboxypropyl)dimethylsilyl]oxy}dimethylsilyl)butanoato-κ2O:O'] perchlorate] (II) Data collection

**Table:**

Agilent Xcalibur, Eos diffractometer	9606 independent reflections
Radiation source: Enhance (Mo) X-ray Source	5769 reflections with *I* > 2σ(*I*)
Graphite monochromator	*R*_int_ = 0.063
Detector resolution: 16.1593 pixels mm^-1^	θ_max_ = 25.0°, θ_min_ = 2.0°
ω scans	*h* = −11→11
Absorption correction: multi-scan (CrysAlis PRO; Agilent, 2014)	*k* = −15→15
*T*_min_ = 0.889, *T*_max_ = 1.000	*l* = −17→17
9606 measured reflections	

### catena-Poly[[[(1,4,8,11-tetraazacyclotetradecane-κ4N1,N4,N8,N11)nickel(II)]-µ-4-({[(3-carboxypropyl)dimethylsilyl]oxy}dimethylsilyl)butanoato-κ2O:O'] perchlorate] (II) Refinement

**Table:**

Refinement on *F*^2^	Primary atom site location: structure-invariant direct methods
Least-squares matrix: full	Hydrogen site location: mixed
*R*[*F*^2^ > 2σ(*F*^2^)] = 0.050	H-atom parameters constrained
*wR*(*F*^2^) = 0.115	*w* = 1/[σ^2^(*F*_o_^2^) + (0.0451*P*)^2^] where *P* = (*F*_o_^2^ + 2*F*_c_^2^)/3
*S* = 1.01	(Δ/σ)_max_ = 0.002
9606 reflections	Δρ_max_ = 0.51 e Å^−^^3^
367 parameters	Δρ_min_ = −0.44 e Å^−^^3^
7 restraints	

### catena-Poly[[[(1,4,8,11-tetraazacyclotetradecane-κ4N1,N4,N8,N11)nickel(II)]-µ-4-({[(3-carboxypropyl)dimethylsilyl]oxy}dimethylsilyl)butanoato-κ2O:O'] perchlorate] (II) Special details

**Table:**

Geometry. All esds (except the esd in the dihedral angle between two l.s. planes) are estimated using the full covariance matrix. The cell esds are taken into account individually in the estimation of esds in distances, angles and torsion angles; correlations between esds in cell parameters are only used when they are defined by crystal symmetry. An approximate (isotropic) treatment of cell esds is used for estimating esds involving l.s. planes.
Refinement. Refined as a 2-component twin.

### catena-Poly[[[(1,4,8,11-tetraazacyclotetradecane-κ4N1,N4,N8,N11)nickel(II)]-µ-4-({[(3-carboxypropyl)dimethylsilyl]oxy}dimethylsilyl)butanoato-κ2O:O'] perchlorate] (II) Fractional atomic coordinates and isotropic or equivalent isotropic displacement parameters (Å^2^)

**Table:**

	*x*	*y*	*z*	*U*_iso_*/*U*_eq_	Occ. (<1)
Ni1	0.000000	0.000000	0.000000	0.0241 (2)	
Si1	−0.3977 (2)	−0.55987 (11)	−0.05469 (15)	0.0729 (6)	
O1	−0.1184 (3)	−0.1542 (2)	−0.0727 (2)	0.0310 (8)	
O2	−0.0826 (3)	−0.1583 (2)	−0.2165 (2)	0.0339 (8)	
H2C	−0.034950	−0.196653	−0.243367	0.051*	0.5
O3	−0.5535 (8)	−0.5084 (6)	−0.0332 (5)	0.061 (3)	0.5
N1	0.1859 (4)	−0.0236 (3)	−0.0499 (3)	0.0408 (11)	
H1	0.157126	−0.087684	−0.099864	0.049*	
N2	−0.0809 (4)	0.0725 (3)	−0.1075 (3)	0.0390 (11)	
H2	−0.128011	0.014955	−0.161639	0.047*	
C1	0.2858 (6)	−0.0494 (5)	0.0295 (4)	0.065 (2)	
H1A	0.335337	0.015969	0.073702	0.078*	
H1B	0.360480	−0.083840	0.006336	0.078*	
C2	−0.1992 (7)	0.1219 (4)	−0.0773 (4)	0.0649 (19)	
H2A	−0.156944	0.189771	−0.034619	0.078*	
H2B	−0.263674	0.135605	−0.131369	0.078*	
C3	0.0282 (7)	0.1448 (4)	−0.1388 (4)	0.0634 (18)	
H3A	0.071451	0.206181	−0.088446	0.076*	
H3B	−0.020855	0.170724	−0.191801	0.076*	
C4	0.1501 (7)	0.0909 (4)	−0.1671 (4)	0.068 (2)	
H4A	0.104405	0.025115	−0.211911	0.081*	
H4B	0.205970	0.137198	−0.199462	0.081*	
C5	0.2570 (6)	0.0641 (4)	−0.0893 (4)	0.0608 (18)	
H5A	0.339987	0.043288	−0.113487	0.073*	
H5B	0.294711	0.127196	−0.039965	0.073*	
C6	−0.1332 (4)	−0.2035 (3)	−0.1537 (3)	0.0254 (11)	
C7	−0.2146 (5)	−0.3171 (3)	−0.1832 (3)	0.0378 (13)	
H7A	−0.303379	−0.319448	−0.229384	0.045*	
H7B	−0.153320	−0.358866	−0.214036	0.045*	
C8	−0.2580 (5)	−0.3700 (3)	−0.1066 (3)	0.0363 (12)	
H8A	−0.316658	−0.327704	−0.073869	0.044*	
H8B	−0.169508	−0.371782	−0.061703	0.044*	
C9	−0.3463 (5)	−0.4842 (3)	−0.1431 (3)	0.0477 (14)	
H9A	−0.289182	−0.524186	−0.179202	0.057*	
H9B	−0.436249	−0.480898	−0.185840	0.057*	
C10	−0.2275 (11)	−0.5710 (6)	0.0258 (5)	0.168 (4)	
H10A	−0.191865	−0.505635	0.071407	0.252*	
H10B	−0.153386	−0.583786	−0.009580	0.252*	
H10C	−0.248872	−0.629417	0.057238	0.252*	
C11	−0.5003 (7)	−0.6957 (4)	−0.1124 (5)	0.102 (3)	
H11A	−0.442542	−0.730299	−0.150911	0.153*	
H11B	−0.592187	−0.690955	−0.150829	0.153*	
H11C	−0.519460	−0.736539	−0.065495	0.153*	
Ni2	0.000000	−0.500000	−0.500000	0.0281 (2)	
Si2	0.35316 (15)	0.04647 (10)	−0.52679 (11)	0.0404 (4)	
O4	0.0305 (3)	−0.3297 (2)	−0.47284 (19)	0.0333 (8)	
O5	−0.0840 (3)	−0.2890 (2)	−0.3576 (2)	0.0376 (9)	
H5C	−0.095950	−0.235276	−0.324118	0.056*	0.5
N3	−0.1442 (6)	−0.5206 (3)	−0.4129 (4)	0.0621 (15)	
H3	−0.143842	−0.449452	−0.377416	0.075*	
N4	0.1844 (5)	−0.4870 (3)	−0.3968 (4)	0.0675 (16)	
H4	0.207815	−0.412879	−0.361974	0.081*	
C12	−0.2937 (7)	−0.5585 (5)	−0.4771 (6)	0.094 (3)	
H12A	−0.370162	−0.545605	−0.442664	0.113*	
H12B	−0.309077	−0.634809	−0.501474	0.113*	
C13	0.3020 (7)	−0.5012 (5)	−0.4435 (7)	0.111 (4)	
H13A	0.295115	−0.576820	−0.467592	0.133*	
H13B	0.395995	−0.474737	−0.399517	0.133*	
C14	0.1635 (10)	−0.5587 (5)	−0.3256 (6)	0.112 (3)	
H14A	0.151266	−0.632881	−0.356424	0.134*	
H14B	0.251865	−0.542354	−0.276130	0.134*	
C15	0.0318 (15)	−0.5454 (6)	−0.2821 (5)	0.136 (4)	
H15A	0.035328	−0.582999	−0.230129	0.163*	
H15B	0.040102	−0.470013	−0.256570	0.163*	
C16	−0.1145 (11)	−0.5851 (5)	−0.3474 (6)	0.116 (4)	
H16A	−0.117774	−0.657098	−0.380421	0.139*	
H16B	−0.191132	−0.588632	−0.311103	0.139*	
C17	0.0036 (5)	−0.2625 (3)	−0.4108 (3)	0.0278 (11)	
C18	0.0724 (5)	−0.1463 (3)	−0.3978 (3)	0.0287 (12)	
H18A	0.132997	−0.123812	−0.334828	0.034*	
H18B	−0.006193	−0.105610	−0.401545	0.034*	
C19	0.1664 (5)	−0.1164 (3)	−0.4662 (3)	0.0359 (12)	
H19A	0.108087	−0.140813	−0.529665	0.043*	
H19B	0.249045	−0.153188	−0.460348	0.043*	
C20	0.2255 (5)	0.0035 (3)	−0.4507 (3)	0.0373 (13)	
H20A	0.276718	0.028112	−0.385648	0.045*	
H20B	0.142064	0.039091	−0.460546	0.045*	
C21	0.2725 (6)	−0.0094 (4)	−0.6529 (4)	0.0706 (18)	
H21A	0.180766	0.013540	−0.670484	0.106*	
H21B	0.255162	−0.086153	−0.663382	0.106*	
H21C	0.339876	0.015653	−0.690111	0.106*	
C22	0.3997 (6)	0.1939 (4)	−0.5079 (5)	0.084 (2)	
H22A	0.445438	0.222461	−0.443122	0.126*	
H22B	0.311213	0.221370	−0.523912	0.126*	
H22C	0.466709	0.214650	−0.546789	0.126*	
O6X	0.493 (3)	−0.013 (5)	−0.497 (6)	0.037 (3)*	0.25
O6	0.510 (3)	0.021 (4)	−0.4772 (12)	0.037 (3)*	0.25
Cl1	0.38292 (17)	−0.21590 (12)	−0.21055 (12)	0.0683 (5)	
O7	0.3941 (6)	−0.2883 (5)	−0.2826 (4)	0.194 (3)	
O8	0.2362 (5)	−0.2323 (4)	−0.1955 (4)	0.1227 (19)	
O9	0.4112 (6)	−0.1102 (4)	−0.2279 (4)	0.147 (2)	
O10	0.4768 (6)	−0.2161 (4)	−0.1271 (4)	0.137 (2)	

### catena-Poly[[[(1,4,8,11-tetraazacyclotetradecane-κ4N1,N4,N8,N11)nickel(II)]-µ-4-({[(3-carboxypropyl)dimethylsilyl]oxy}dimethylsilyl)butanoato-κ2O:O'] perchlorate] (II) Atomic displacement parameters (Å^2^)

**Table:**

	*U*^11^	*U*^22^	*U*^33^	*U*^12^	*U*^13^	*U*^23^
Ni1	0.0254 (4)	0.0202 (4)	0.0226 (5)	−0.0021 (4)	0.0072 (4)	−0.0040 (3)
Si1	0.1134 (15)	0.0274 (8)	0.0967 (15)	0.0056 (9)	0.0774 (14)	0.0135 (8)
O1	0.0404 (19)	0.0256 (16)	0.0206 (19)	−0.0064 (14)	0.0113 (15)	−0.0065 (14)
O2	0.051 (2)	0.0206 (16)	0.030 (2)	−0.0038 (14)	0.0228 (17)	−0.0013 (14)
O3	0.085 (7)	0.051 (4)	0.069 (8)	0.020 (5)	0.056 (5)	0.020 (5)
N1	0.033 (2)	0.038 (2)	0.041 (3)	−0.005 (2)	0.016 (2)	−0.017 (2)
N2	0.052 (3)	0.026 (2)	0.031 (3)	0.006 (2)	−0.001 (2)	−0.0044 (18)
C1	0.028 (3)	0.065 (4)	0.081 (5)	0.019 (3)	−0.006 (3)	−0.032 (4)
C2	0.072 (4)	0.048 (3)	0.058 (4)	0.024 (3)	−0.022 (4)	−0.009 (3)
C3	0.119 (5)	0.027 (3)	0.032 (3)	−0.010 (3)	0.006 (4)	0.003 (2)
C4	0.106 (5)	0.045 (3)	0.036 (4)	−0.041 (3)	0.039 (4)	−0.009 (3)
C5	0.059 (4)	0.060 (4)	0.051 (4)	−0.023 (3)	0.036 (3)	−0.018 (3)
C6	0.026 (3)	0.025 (2)	0.023 (3)	0.000 (2)	0.008 (2)	−0.002 (2)
C7	0.053 (3)	0.027 (3)	0.027 (3)	−0.010 (2)	0.018 (3)	−0.004 (2)
C8	0.048 (3)	0.025 (2)	0.033 (3)	−0.001 (2)	0.016 (3)	−0.002 (2)
C9	0.056 (3)	0.028 (3)	0.053 (4)	−0.010 (2)	0.022 (3)	−0.004 (2)
C10	0.274 (12)	0.096 (6)	0.091 (7)	−0.042 (7)	−0.030 (7)	0.047 (5)
C11	0.075 (5)	0.042 (4)	0.192 (8)	−0.006 (3)	0.039 (5)	0.034 (4)
Ni2	0.0285 (5)	0.0228 (4)	0.0292 (5)	−0.0009 (4)	0.0125 (4)	−0.0067 (4)
Si2	0.0330 (8)	0.0396 (8)	0.0503 (10)	0.0047 (7)	0.0090 (7)	0.0156 (7)
O4	0.0421 (19)	0.0242 (16)	0.0334 (19)	0.0031 (14)	0.0212 (16)	−0.0061 (14)
O5	0.053 (2)	0.0263 (17)	0.033 (2)	−0.0013 (15)	0.0258 (18)	−0.0061 (14)
N3	0.099 (4)	0.026 (2)	0.073 (4)	0.007 (3)	0.064 (3)	−0.002 (3)
N4	0.056 (3)	0.043 (3)	0.080 (4)	0.016 (3)	−0.019 (3)	−0.027 (3)
C12	0.049 (4)	0.056 (4)	0.167 (7)	−0.010 (3)	0.066 (5)	−0.033 (4)
C13	0.039 (4)	0.062 (5)	0.188 (10)	0.014 (4)	−0.027 (5)	−0.048 (5)
C14	0.164 (8)	0.055 (4)	0.088 (6)	0.040 (5)	−0.055 (6)	0.004 (4)
C15	0.313 (15)	0.067 (5)	0.049 (5)	0.084 (8)	0.047 (8)	0.019 (4)
C16	0.238 (11)	0.045 (4)	0.088 (7)	0.012 (6)	0.122 (7)	0.001 (4)
C17	0.028 (3)	0.028 (2)	0.024 (3)	0.005 (2)	0.003 (2)	0.000 (2)
C18	0.033 (3)	0.018 (2)	0.036 (3)	0.003 (2)	0.014 (2)	0.001 (2)
C19	0.038 (3)	0.031 (3)	0.041 (3)	0.004 (2)	0.017 (3)	0.003 (2)
C20	0.034 (3)	0.035 (3)	0.041 (3)	0.003 (2)	0.011 (3)	0.002 (2)
C21	0.079 (4)	0.088 (4)	0.045 (4)	0.017 (4)	0.011 (3)	0.017 (3)
C22	0.082 (5)	0.048 (3)	0.116 (6)	−0.012 (3)	0.023 (4)	0.021 (4)
Cl1	0.0571 (10)	0.0599 (10)	0.0695 (12)	0.0139 (8)	−0.0151 (10)	−0.0111 (9)
O7	0.137 (5)	0.163 (5)	0.202 (6)	−0.019 (4)	0.037 (4)	−0.146 (5)
O8	0.077 (3)	0.154 (5)	0.144 (5)	0.026 (3)	0.017 (3)	0.050 (4)
O9	0.176 (6)	0.079 (4)	0.158 (5)	−0.006 (4)	−0.005 (5)	0.016 (3)
O10	0.117 (4)	0.143 (4)	0.115 (4)	0.037 (3)	−0.050 (4)	−0.006 (4)

### catena-Poly[[[(1,4,8,11-tetraazacyclotetradecane-κ4N1,N4,N8,N11)nickel(II)]-µ-4-({[(3-carboxypropyl)dimethylsilyl]oxy}dimethylsilyl)butanoato-κ2O:O'] perchlorate] (II) Geometric parameters (Å, º)

**Table:**

Ni1—O1	2.125 (2)	Ni2—N4	2.054 (4)
Ni1—O1^i^	2.125 (2)	Ni2—N4^iii^	2.054 (4)
Ni1—N1^i^	2.058 (3)	Si2—C20	1.864 (5)
Ni1—N1	2.058 (3)	Si2—C21	1.855 (5)
Ni1—N2^i^	2.060 (4)	Si2—C22	1.845 (5)
Ni1—N2	2.060 (4)	Si2—O6X^iv^	1.570 (19)
Si1—O3	1.757 (8)	Si2—O6X	1.651 (19)
Si1—O3^ii^	1.626 (7)	Si2—O6^iv^	1.66 (2)
Si1—C9	1.837 (5)	Si2—O6	1.632 (16)
Si1—C10	1.852 (9)	O4—C17	1.245 (4)
Si1—C11	1.845 (5)	O5—H5C	0.8199
O1—C6	1.232 (4)	O5—C17	1.280 (5)
O2—H2C	0.8200	N3—H3	0.9800
O2—C6	1.291 (5)	N3—C12	1.502 (8)
O3—O3^ii^	1.234 (13)	N3—C16	1.393 (9)
N1—H1	0.9800	N4—H4	0.9800
N1—C1	1.481 (6)	N4—C13	1.422 (8)
N1—C5	1.475 (6)	N4—C14	1.527 (9)
N2—H2	0.9800	C12—H12A	0.9700
N2—C2	1.467 (6)	C12—H12B	0.9700
N2—C3	1.461 (6)	C12—C13^iii^	1.502 (10)
C1—H1A	0.9700	C13—H13A	0.9700
C1—H1B	0.9700	C13—H13B	0.9700
C1—C2^i^	1.486 (7)	C14—H14A	0.9700
C2—H2B	0.9700	C14—H14B	0.9700
C2—H2A	0.9700	C14—C15	1.512 (11)
C3—H3A	0.9700	C15—H15A	0.9700
C3—H3B	0.9700	C15—H15B	0.9700
C3—C4	1.516 (7)	C15—C16	1.488 (11)
C4—H4A	0.9700	C16—H16A	0.9700
C4—H4B	0.9700	C16—H16B	0.9700
C4—C5	1.507 (7)	C20—H20A	0.9700
C5—H5A	0.9700	C20—H20B	0.9700
C5—H5B	0.9700	C20—C19	1.521 (5)
C6—C7	1.496 (5)	C19—H19A	0.9700
C7—H7A	0.9700	C19—H19B	0.9700
C7—H7B	0.9700	C19—C18	1.512 (6)
C7—C8	1.496 (6)	C18—H18A	0.9700
C8—H8A	0.9700	C18—H18B	0.9700
C8—H8B	0.9700	C18—C17	1.501 (5)
C8—C9	1.529 (5)	C21—H21A	0.9600
C9—H9A	0.9700	C21—H21B	0.9600
C9—H9B	0.9700	C21—H21C	0.9600
C10—H10A	0.9600	C22—H22A	0.9600
C10—H10B	0.9600	C22—H22B	0.9600
C10—H10C	0.9600	C22—H22C	0.9600
C11—H11A	0.9600	O6X—O6X^iv^	0.38 (7)
C11—H11B	0.9600	O6—O6^iv^	0.77 (5)
C11—H11C	0.9600	Cl1—O7	1.329 (4)
Ni2—O4^iii^	2.131 (2)	Cl1—O8	1.421 (5)
Ni2—O4	2.131 (2)	Cl1—O9	1.420 (5)
Ni2—N3^iii^	2.043 (4)	Cl1—O10	1.380 (5)
Ni2—N3	2.043 (4)		
			
O1—Ni1—O1^i^	180.0	N3^iii^—Ni2—N4	85.7 (2)
N1^i^—Ni1—O1	88.17 (12)	N4—Ni2—O4^iii^	91.33 (14)
N1^i^—Ni1—O1^i^	91.83 (12)	N4—Ni2—O4	88.67 (14)
N1—Ni1—O1^i^	88.17 (12)	N4^iii^—Ni2—O4	91.33 (14)
N1—Ni1—O1	91.83 (12)	N4^iii^—Ni2—O4^iii^	88.67 (14)
N1^i^—Ni1—N1	180.0	N4—Ni2—N4^iii^	180.0
N1^i^—Ni1—N2	85.82 (17)	C21—Si2—C20	111.6 (2)
N1—Ni1—N2^i^	85.82 (17)	C22—Si2—C20	110.6 (2)
N1^i^—Ni1—N2^i^	94.18 (17)	C22—Si2—C21	109.6 (3)
N1—Ni1—N2	94.18 (17)	O6X^iv^—Si2—C20	113.4 (14)
N2—Ni1—O1^i^	87.38 (12)	O6X—Si2—C20	102.5 (13)
N2^i^—Ni1—O1	87.38 (12)	O6X—Si2—C21	107 (3)
N2—Ni1—O1	92.62 (12)	O6X^iv^—Si2—C21	107 (3)
N2^i^—Ni1—O1^i^	92.62 (12)	O6X—Si2—C22	116 (2)
N2—Ni1—N2^i^	180.0	O6X^iv^—Si2—C22	104 (2)
O3^ii^—Si1—O3	42.6 (4)	O6X^iv^—Si2—O6X	13 (3)
O3^ii^—Si1—C9	115.6 (3)	O6X—Si2—O6^iv^	13 (3)
O3—Si1—C9	100.2 (3)	O6X^iv^—Si2—O6^iv^	17 (2)
O3—Si1—C10	131.5 (4)	O6^iv^—Si2—C20	111.2 (17)
O3^ii^—Si1—C10	89.4 (4)	O6—Si2—C20	103.5 (15)
O3^ii^—Si1—C11	121.3 (4)	O6—Si2—C21	120.4 (6)
O3—Si1—C11	95.7 (3)	O6^iv^—Si2—C21	94.3 (5)
C9—Si1—C10	109.0 (3)	O6—Si2—C22	100.3 (19)
C9—Si1—C11	110.1 (3)	O6^iv^—Si2—C22	118.5 (19)
C11—Si1—C10	108.8 (3)	O6—Si2—O6^iv^	26.9 (17)
C6—O1—Ni1	132.9 (3)	C17—O4—Ni2	133.8 (3)
C6—O2—H2C	109.8	C17—O5—H5C	109.9
Si1^ii^—O3—Si1	137.4 (4)	Ni2—N3—H3	106.9
O3^ii^—O3—Si1	63.0 (6)	C12—N3—Ni2	105.1 (4)
O3^ii^—O3—Si1^ii^	74.4 (7)	C12—N3—H3	106.9
Ni1—N1—H1	107.4	C16—N3—Ni2	117.3 (4)
C1—N1—Ni1	105.0 (3)	C16—N3—H3	106.9
C1—N1—H1	107.4	C16—N3—C12	113.2 (6)
C5—N1—Ni1	116.2 (3)	Ni2—N4—H4	106.8
C5—N1—H1	107.4	C13—N4—Ni2	106.5 (4)
C5—N1—C1	113.1 (4)	C13—N4—H4	106.8
Ni1—N2—H2	106.7	C13—N4—C14	115.0 (6)
C2—N2—Ni1	105.7 (3)	C14—N4—Ni2	114.4 (4)
C2—N2—H2	106.7	C14—N4—H4	106.8
C3—N2—Ni1	116.1 (3)	N3—C12—H12A	109.9
C3—N2—H2	106.7	N3—C12—H12B	109.9
C3—N2—C2	114.2 (4)	N3—C12—C13^iii^	108.8 (5)
N1—C1—H1A	109.7	H12A—C12—H12B	108.3
N1—C1—H1B	109.7	C13^iii^—C12—H12A	109.9
N1—C1—C2^i^	109.7 (4)	C13^iii^—C12—H12B	109.9
H1A—C1—H1B	108.2	N4—C13—C12^iii^	109.6 (6)
C2^i^—C1—H1A	109.7	N4—C13—H13A	109.8
C2^i^—C1—H1B	109.7	N4—C13—H13B	109.8
N2—C2—C1^i^	109.8 (4)	C12^iii^—C13—H13A	109.8
N2—C2—H2B	109.7	C12^iii^—C13—H13B	109.8
N2—C2—H2A	109.7	H13A—C13—H13B	108.2
C1^i^—C2—H2B	109.7	N4—C14—H14A	109.0
C1^i^—C2—H2A	109.7	N4—C14—H14B	109.0
H2B—C2—H2A	108.2	H14A—C14—H14B	107.8
N2—C3—H3A	109.1	C15—C14—N4	113.0 (6)
N2—C3—H3B	109.1	C15—C14—H14A	109.0
N2—C3—C4	112.3 (4)	C15—C14—H14B	109.0
H3A—C3—H3B	107.9	C14—C15—H15A	108.5
C4—C3—H3A	109.1	C14—C15—H15B	108.5
C4—C3—H3B	109.1	H15A—C15—H15B	107.5
C3—C4—H4A	108.1	C16—C15—C14	115.0 (6)
C3—C4—H4B	108.1	C16—C15—H15A	108.5
H4A—C4—H4B	107.3	C16—C15—H15B	108.5
C5—C4—C3	116.7 (4)	N3—C16—C15	113.1 (7)
C5—C4—H4A	108.1	N3—C16—H16A	109.0
C5—C4—H4B	108.1	N3—C16—H16B	109.0
N1—C5—C4	111.4 (4)	C15—C16—H16A	109.0
N1—C5—H5A	109.4	C15—C16—H16B	109.0
N1—C5—H5B	109.4	H16A—C16—H16B	107.8
C4—C5—H5A	109.4	Si2—C20—H20A	108.3
C4—C5—H5B	109.4	Si2—C20—H20B	108.3
H5A—C5—H5B	108.0	H20A—C20—H20B	107.4
O1—C6—O2	121.4 (4)	C19—C20—Si2	115.9 (3)
O1—C6—C7	120.8 (4)	C19—C20—H20A	108.3
O2—C6—C7	117.7 (4)	C19—C20—H20B	108.3
C6—C7—H7A	108.3	C20—C19—H19A	109.0
C6—C7—H7B	108.3	C20—C19—H19B	109.0
H7A—C7—H7B	107.4	H19A—C19—H19B	107.8
C8—C7—C6	116.0 (4)	C18—C19—C20	113.1 (3)
C8—C7—H7A	108.3	C18—C19—H19A	109.0
C8—C7—H7B	108.3	C18—C19—H19B	109.0
C7—C8—H8A	109.0	C19—C18—H18A	108.1
C7—C8—H8B	109.0	C19—C18—H18B	108.1
C7—C8—C9	112.8 (4)	H18A—C18—H18B	107.3
H8A—C8—H8B	107.8	C17—C18—C19	116.7 (3)
C9—C8—H8A	109.0	C17—C18—H18A	108.1
C9—C8—H8B	109.0	C17—C18—H18B	108.1
Si1—C9—H9A	108.1	O4—C17—O5	121.9 (4)
Si1—C9—H9B	108.1	O4—C17—C18	120.1 (4)
C8—C9—Si1	116.8 (3)	O5—C17—C18	118.0 (3)
C8—C9—H9A	108.1	Si2—C21—H21A	109.5
C8—C9—H9B	108.1	Si2—C21—H21B	109.5
H9A—C9—H9B	107.3	Si2—C21—H21C	109.5
Si1—C10—H10A	109.5	H21A—C21—H21B	109.5
Si1—C10—H10B	109.5	H21A—C21—H21C	109.5
Si1—C10—H10C	109.5	H21B—C21—H21C	109.5
H10A—C10—H10B	109.5	Si2—C22—H22A	109.5
H10A—C10—H10C	109.5	Si2—C22—H22B	109.5
H10B—C10—H10C	109.5	Si2—C22—H22C	109.5
Si1—C11—H11A	109.5	H22A—C22—H22B	109.5
Si1—C11—H11B	109.5	H22A—C22—H22C	109.5
Si1—C11—H11C	109.5	H22B—C22—H22C	109.5
H11A—C11—H11B	109.5	Si2^iv^—O6X—Si2	167 (3)
H11A—C11—H11C	109.5	O6X^iv^—O6X—Si2^iv^	96 (6)
H11B—C11—H11C	109.5	O6X^iv^—O6X—Si2	71 (6)
O4—Ni2—O4^iii^	180.00 (3)	Si2—O6—Si2^iv^	153.1 (17)
N3^iii^—Ni2—O4^iii^	94.96 (13)	O6^iv^—O6—Si2	78 (2)
N3—Ni2—O4	94.96 (13)	O7—Cl1—O8	110.3 (3)
N3^iii^—Ni2—O4	85.04 (13)	O7—Cl1—O9	112.0 (4)
N3—Ni2—O4^iii^	85.04 (13)	O7—Cl1—O10	114.2 (4)
N3^iii^—Ni2—N3	180.0	O9—Cl1—O8	105.3 (3)
N3^iii^—Ni2—N4^iii^	94.3 (2)	O10—Cl1—O8	107.7 (4)
N3—Ni2—N4	94.3 (2)	O10—Cl1—O9	106.8 (3)
N3—Ni2—N4^iii^	85.7 (2)		
			
Ni1—O1—C6—O2	−7.2 (6)	Ni2—N4—C14—C15	−52.9 (7)
Ni1—O1—C6—C7	174.5 (3)	Si2—C20—C19—C18	176.0 (3)
Ni1—N1—C1—C2^i^	40.9 (4)	N4—C14—C15—C16	68.4 (9)
Ni1—N1—C5—C4	−56.2 (5)	C12—N3—C16—C15	−178.2 (6)
Ni1—N2—C2—C1^i^	−39.8 (4)	C13—N4—C14—C15	−176.6 (6)
Ni1—N2—C3—C4	55.3 (5)	C14—N4—C13—C12^iii^	169.9 (5)
O1—C6—C7—C8	−7.7 (6)	C14—C15—C16—N3	−71.7 (8)
O2—C6—C7—C8	174.0 (4)	C16—N3—C12—C13^iii^	−168.2 (5)
O3^ii^—Si1—O3—Si1^ii^	−0.001 (2)	C20—Si2—O6X—Si2^iv^	−148 (28)
O3—Si1—C9—C8	81.0 (4)	C20—Si2—O6X—O6X^iv^	−148 (28)
O3^ii^—Si1—C9—C8	39.0 (5)	C20—Si2—O6—Si2^iv^	110 (7)
N2—C3—C4—C5	−70.0 (6)	C20—Si2—O6—O6^iv^	110 (7)
C1—N1—C5—C4	−177.7 (4)	C20—C19—C18—C17	177.2 (4)
C2—N2—C3—C4	178.7 (4)	C19—C18—C17—O4	3.3 (6)
C3—N2—C2—C1^i^	−168.7 (4)	C19—C18—C17—O5	−175.7 (4)
C3—C4—C5—N1	70.1 (5)	C21—Si2—C20—C19	52.0 (4)
C5—N1—C1—C2^i^	168.6 (4)	C21—Si2—O6X—Si2^iv^	95 (29)
C6—C7—C8—C9	177.5 (4)	C21—Si2—O6X—O6X^iv^	95 (29)
C7—C8—C9—Si1	176.9 (4)	C21—Si2—O6—Si2^iv^	−15 (9)
C9—Si1—O3—Si1^ii^	−116.9 (7)	C21—Si2—O6—O6^iv^	−15 (9)
C9—Si1—O3—O3^ii^	−116.9 (7)	C22—Si2—C20—C19	174.3 (4)
C10—Si1—O3—Si1^ii^	10.0 (10)	C22—Si2—O6X—Si2^iv^	−27 (30)
C10—Si1—O3—O3^ii^	10.0 (10)	C22—Si2—O6X—O6X^iv^	−27 (30)
C10—Si1—C9—C8	−59.7 (5)	C22—Si2—O6—Si2^iv^	−135 (8)
C11—Si1—O3—Si1^ii^	131.5 (8)	C22—Si2—O6—O6^iv^	−135 (8)
C11—Si1—O3—O3^ii^	131.5 (8)	O6X^iv^—Si2—C20—C19	−70 (4)
C11—Si1—C9—C8	−179.0 (4)	O6X—Si2—C20—C19	−62 (3)
Ni2—O4—C17—O5	−16.3 (6)	O6X^iv^—Si2—O6X—Si2^iv^	−0.01 (14)
Ni2—O4—C17—C18	164.8 (3)	O6^iv^—Si2—C20—C19	−51.9 (12)
Ni2—N3—C12—C13^iii^	−39.0 (6)	O6—Si2—C20—C19	−79.0 (15)
Ni2—N3—C16—C15	59.1 (7)	O6^iv^—Si2—O6—Si2^iv^	0.006 (14)
Ni2—N4—C13—C12^iii^	42.1 (6)		

Symmetry codes: (i) −*x*, −*y*, −*z*; (ii) −*x*−1, −*y*−1, −*z*; (iii) −*x*, −*y*−1, −*z*−1; (iv) −*x*+1, −*y*, −*z*−1.

### catena-Poly[[[(1,4,8,11-tetraazacyclotetradecane-κ4N1,N4,N8,N11)nickel(II)]-µ-4-({[(3-carboxypropyl)dimethylsilyl]oxy}dimethylsilyl)butanoato-κ2O:O'] perchlorate] (II) Hydrogen-bond geometry (Å, º)

**Table:**

*D*—H···*A*	*D*—H	H···*A*	*D*···*A*	*D*—H···*A*
N1—H1···O2	0.98	2.51	3.225 (5)	130
N1—H1···O8	0.98	2.45	3.315 (6)	147
N2—H2···O2	0.98	2.38	3.143 (4)	134
N3—H3···O5	0.98	2.01	2.901 (5)	150
N4—H4···O7	0.98	2.18	3.012 (6)	142
O2—H2*C*···O5	0.82	1.84	2.456 (4)	131
O2—H2*C*···O8	0.82	2.65	3.260 (5)	133
O5—H5*C*···O2	0.82	1.70	2.456 (4)	151
